# EHMTI-0274. Secondary headache due to orbital pseudotumor: a case report

**DOI:** 10.1186/1129-2377-15-S1-C14

**Published:** 2014-09-18

**Authors:** S Kutluhan, H Degirmenci, M Sahin, B Kale

**Affiliations:** 1 School of Medicine, Süleyman Demirel University, Isparta, Turkey

## 

We present a case report in which a secondary headache caused by orbital pseudotumor (OP) that has been treated with high doses of steroids.

A female patient, 44 years old, who complained having 12 years of headache. Last five years it had been very acute and compulsive. This was especially on the left side of her head, and it severely effected her left eye. Her left eye and its eyelash were swollen. The severity of headache was increased with movements. There was no autonomic finding. Third and sixth cranial nerve palsy and optic atrophy (OA) developed in her left eye. The patient had diabetes insipidus and diabetes mellitus. The diagnosis of the patient considered the DIDMOAD syndrome without deafness. The severity of the headache was controlled and reduced by steroid medication. Analgesics and antiepileptics didn’t help.

In the clinical history of case; hypothyroidism and ankylosing spondylitis were present. The physical examination of case was revealed mydriasis, light responsiveness, proptosis, and movement restriction of in all direction in the left eye, and OA. Orbital MRI showed the OP on the left eye, but the etiology remains unexplained. The patient's complaint was decreased seven times pulse form to 1000 mg per day by giving prednisolone. After this treatment the prednisolone dosage was reduced from 1000 mg to 250 mg per day. Rituximab 1000 mg per 15 day were given to the patient. Recently, the patient has complained with a pain started in the right eye. Left ocular muscle biopsy was performed.

No conflict of interest.

